# Analysis of Adherence Status and Influencing Factors Among Middle-Aged and Elderly Hypertension Patients in Rural Areas of Northeast China

**DOI:** 10.1155/ijhy/9954099

**Published:** 2025-04-26

**Authors:** Xinyuan Lu, Jiwei Wang, Sikun Chen, Lin Lv, Jinming Yu

**Affiliations:** Key Laboratory of Public Health Safety Ministry of Education, National Health Commission Key Laboratory of Health Technology Assessment, School of Public Health, Fudan University, Shanghai 200043, China

**Keywords:** behavior adherence, dietary adherence, hypertension, medication adherence, middle-aged and elderly adults

## Abstract

Hypertension remains a major public health challenge globally, with suboptimal adherence to treatment and lifestyle modifications exacerbating cardiovascular risks. This study evaluates multidimensional adherence (medication, diet, and behavior) and its determinants among hypertensive patients in rural Northeast China. A cross-sectional study enrolled 6352 adults aged ≥ 40 years with diagnosed and poorly controlled hypertension from rural villages across five cities (Benxi, Chaoyang, Dandong, Donggang, and Fuxin) in Liaoning Province, Northeast China, using multistage cluster sampling. Adherence was assessed via standardized questionnaires, with logistic regression analyzing sociodemographic, clinical, and behavioral predictors. Medication adherence was reported by 73.7% of participants, while dietary and behavioral adherence rates were 10.5% and 29.3%, respectively. Ethnic disparities emerged, with Han Chinese exhibiting lower medication adherence (aOR = 0.485, 95% CI: 0.377–0.624). Cohabiting with children enhanced dietary adherence (aOR = 2.184, 95% CI: 1.854–2.573), whereas widowed status reduced both dietary (aOR = 0.698, 95% CI: 0.528–0.924) and behavioral adherence (aOR = 0.726, 95% CI: 0.595–0.887). Higher hypertension knowledge scores positively influenced all adherence domains (*p* < 0.05). Adherence among rural hypertensive patients is multidimensional, shaped by cultural, socioeconomic, and behavioral factors. Targeted interventions addressing dietary sodium reduction, family-based support, and health literacy improvement are urgently needed. This study underscores the importance of integrating region-specific strategies into hypertension management programs to mitigate cardiovascular morbidity in high-risk populations.

## 1. Introduction

According to statistical data, the global population of individuals aged 30–79 years with hypertension surged from 650 million to 1.28 billion between 1990 and 2019 [1]. China is among the countries bearing the most severe global burden of hypertension [2]. As reported in the 2021 Report on Cardiovascular Health and Diseases in China, the number of hypertension patients in China has reached 245 million, reflecting not only a substantial figure but also a continuing upward trend [3]. Despite this large population affected by hypertension, the rates of awareness, treatment, and control of hypertension in China remain suboptimal [4]. Recent data indicate that in 2018, the awareness, treatment, and control rates of hypertension among Chinese adult residents were merely 41%, 34.9%, and 11.0%, respectively [5]. Enhancing hypertension control rates and mitigating cardiovascular diseases and premature deaths attributable to hypertension have thus emerged as critical priorities in global public health efforts [2, 6].

Adherence is crucial for chronic somatic and psychiatric disorders requiring long-term maintenance treatment [7]. The World Health Organization (WHO) defines adherence as “the extent to which a person's behavior-taking medication, following a diet, and/or executing lifestyle changes, corresponds with agreed recommendations from a health care provider” [8]. Improving adherence is of paramount importance for the prevention and management of hypertension. Numerous studies have demonstrated that enhanced adherence is associated with better control of blood pressure levels [9, 10], a reduction in the global burden of disease [11, 12], and a decreased risk of complications such as stroke, myocardial infarction, and heart failure (HF) [13–16].

Despite the critical importance of adherence, the current adherence rates among hypertensive patients remain concerning. A meta-analysis involving 27 million patients revealed that the global prevalence of non-adherence to antihypertensive medication ranges from 27% to 40% [12]. Wen Min et al. conducted a study involving a sample of 44,322 hypertension patients, which indicated that the adherence rate among hypertensive patients in mainland China is only 42.5%. Specifically, the adherence rates in Northeast, East, and Central-West China were 36.9%, 40.6%, and 47.9%, respectively [17]. According to survey data from the National Health and Nutrition Examination Survey (NHANES, 1999–2004) in the United States, only 19.4% of hypertensive patients demonstrated good adherence to the Dietary Approaches to Stop Hypertension (DASH) diet, with the proportion showing a further declining trend [18]. Other studies have found that merely 22.0% of patients continue to choose the DASH diet even after being explicitly diagnosed with hypertension [19]. A nationally representative survey on trends in major non-communicable diseases and related risk factors in China (2002–2019) revealed minimal improvement—and even deterioration—in modifiable behavioral risk factors such as smoking, excessive alcohol consumption, insufficient fruit/vegetable intake, high red meat consumption, and physical inactivity [20]. Furthermore, the analysis of healthy lifestyle patterns in the China Kadoorie Biobank (CKB) cohort demonstrated that only 0.7% of Chinese adults simultaneously adhered to six key health behaviors: nonsmoking, moderate alcohol intake, healthy diet, regular physical activity, normal BMI (18.5–23.9 kg/m^2^), and healthy waist circumference (male < 85 cm; female < 80 cm) [21].

According to the conceptual framework provided by the WHO in 2003 [8], numerous factors can influence adherence to hypertension treatment. These factors include socioeconomic-related factors (e.g., poor socioeconomic status [22], lower education levels, and high medication costs [23]), healthcare team/health system-related factors (e.g., lack of knowledge [24], inadequate time for consultations), condition-related factors (e.g., understanding and perceptions about hypertension [25]), therapy-related factors (e.g., duration of treatment [26], adverse effects of treatment [27]), and patient-related factors (e.g., readiness to change, self-efficacy [28]). However, with the evolution of the global economy, the relationship between some of these factors and hypertension adherence may have become less pronounced, while new influencing factors may have emerged. Recent studies have identified that anxiety and depression may also significantly impact adherence among hypertensive patients [29, 30].

It is noteworthy that the WHO's definition of adherence extends beyond medication adherence to encompass a broad range of health-related behaviors, including but not limited to the use of prescribed pharmaceuticals. Furthermore, from the patient's perspective, adherence requires their active agreement with the recommendations, distinguishing it from the more passive concept of compliance [8]. However, existing research tends to focus on isolated aspects, particularly medication adherence, and lacks a comprehensive analysis of adherence among hypertensive patients. Therefore, this study aims to investigate the adherence status of hypertension patients in Northeast China across three dimensions—medication adherence, dietary adherence, and behavioral adherence—and to explore the factors influencing adherence in this population.

## 2. Materials and Methods

### 2.1. Study Design and Participants

This cross-sectional study was conducted in Liaoning Province, China. Participants were recruited from villages across multiple cities using a multi-stage cluster random sampling method. The inclusion criteria for eligible participants were as follows: (1) age ≥ 40 years; (2) untreated mean systolic blood pressure (SBP) ≥ 140 mmHg and/or diastolic blood pressure (DBP) ≥ 90 mmHg, or treated mean SBP ≥ 130 mmHg and/or DBP ≥ 80 mmHg; treated or untreated mean SBP ≥ 130 mmHg and/or DBP ≥ 80 mmHg in individuals with a clinical history of coronary heart disease (CHD), HF, stroke, diabetes mellitus, or chronic kidney disease (CKD) [4]; (3) established hypertension records in rural clinics; and (4) willingness to participate and ability to provide written informed consent. Participants who were unable to independently complete the questionnaire or lacked hypertension records in village clinics were excluded from the study.

A total of 7362 individuals from 70 villages across five cities (Benxi, Chaoyang, Dandong, Donggang, and Fuxin) in Liaoning Province were enrolled. All participants were administered standardized questionnaires to assess sociodemographic characteristics, clinical history, lifestyle behaviors, and adherence metrics, including medication compliance, dietary habits, and behavioral patterns. Additionally, blood pressure measurements were conducted for each participant. Data were collected anonymously. After excluding subjects with missing adherence or general characteristic information, 6352 participants (86.3%) were included in the final analysis. The study protocol was approved by the Ethics Committee of the First Hospital of China Medical University (Approval No.: [2018]083). Written informed consent was obtained from all participants.

### 2.2. Definition of Variables

#### 2.2.1. Independent Variables

The independent variables in this study comprised age; sex; ethnicity (Han Chinese and others); household composition (categorized as 1, 2, or ≥ 3 permanent members); residential status (classified as living alone/with spouse only, living with children, or other arrangement); educational attainment (stratified as no formal education, primary school, or middle school/higher); marital status (grouped as married/cohabiting, widowed, or divorced/separated/single); smoking status (non-smoker, former smoker, current smoker); alcohol consumption (non-drinker, former drinker, current drinker); pickled food consumption frequency (four-level scale from “never” to “always”); household monthly salt intake; weekly fruit/vegetable consumption (five-level scale from “seldom” to “≥ 4 kg”); physical activity frequency (five-level scale from “never” to “≥ 7 times/week”); anthropometric measurements (weight, height, waist circumference, hip circumference); medical history (major cardiovascular diseases, diabetes, CKD); antihypertensive medication use (categorized as 1, 2, or ≥ 3 daily medications); monthly hypertension-related expenditure (Chinese Yuan, CNY); and hypertension knowledge score. Permanent household members were defined as those cohabiting with participants for > 6 months annually. Body mass index (BMI) was calculated using the standard formula: weight (kg)/[height (m)]^2^, with classification thresholds set at < 18.5 (underweight), 18.5–23.9 (normal range), 24.0–27.9 (overweight), and > 27.9 (obese) [31].

Hypertension knowledge was assessed using an 11-item researcher-developed questionnaire demonstrating good reliability (Cronbach's *α* = 0.826) and validity (KMO = 0.882; Bartlett's sphericity test: *p* < 0.001), evaluating four domains: disease awareness (“Is hypertension a lifelong condition?”; “Is hypertension preventable?”), diagnostic thresholds (“Are you aware of hypertension diagnostic criteria?”), lifestyle management (“Should daily salt intake [including sauces/processed foods] be restricted to < 6 g/day?”; “Does salt restriction/weight control benefit prevention/management?”; “Does smoking/physical inactivity increase risk?”; “Is alcohol control necessary?”), and treatment adherence (“Is regular blood pressure monitoring/long-term medication essential?”), with a binary scoring system (1 point/correct answer; total score range: 0–11).

#### 2.2.2. Dependent Variables

According to the WHO's definition of adherence, this study will explore adherence from three perspectives: medication adherence, dietary adherence, and behavioral adherence. Unfortunately, there is currently no absolute “gold standard” for measuring adherence, as both subjective methods and objective strategies have limitations in assessing adherence [14]. Based on the Chinese Guidelines for the Prevention and Treatment of Hypertension (2024 Revision) and relevant studies [4, 32–34], and taking practical considerations into account, this study ultimately adopts a self-reporting approach to measure adherence. The summarized measures of the three types of adherence are shown in Table 1

### 2.3. Statistical Analysis

Data analyses were conducted using scripts developed in R version 4.2.1 (R Foundation for Statistical Computing, Vienna, Austria), with a significance level set at 0.05. Descriptive statistics were employed to summarize the baseline characteristics and adherence levels of participants. Continuous variables were expressed as means ± standard deviation (SD), while categorical variables were presented as frequencies (percentages), as appropriate. Univariate logistic regression was used to compare adherence differences among participants with varying demographic characteristics, yielding crude odds ratios (cORs). Medication adherence, dietary adherence, and behavioral adherence were designated as dependent variables in separate models. Household monthly salt intake, waist circumference, hip circumference, monthly hypertension-related expenditure, and hypertension knowledge were treated as continuous variables. Forward variable selection was applied to screen variables, the goodness-of-fit of the model was assessed using the Hosmer–Lemeshow test, and the regression coefficients of the model were evaluated using the Wald test. The variance inflation factor (VIF) was calculated to diagnose multicollinearity among the independent variables. Coefficients, adjusted odds ratios (aORs), and *p* values were computed for each variable. Odds ratios were reported with 95% confidence intervals (95% CIs).

## 3. Results

### 3.1. General Characteristics

From May 2019 to January 2020, this cross-sectional study enrolled 6352 middle-aged and older adults diagnosed with hypertension. The study population had a mean age of 62.9 ± 8.8 years, with 44.7% (*n* = 2840) of participants being aged 65 years or older. Additionally, females constituted 62.7% (*n* = 3981) of the cohort. The majority identified as Han Chinese (91.8%, *n* = 5828) and were married or cohabiting (87.3%, *n* = 5545). Educational attainment varied: 51.4% (*n* = 3263) completed primary education, 20.6% (*n* = 1307) reported no formal education, and 28.1% (*n* = 1782) attained middle school or higher. Lifestyle risk factors were prevalent: 19.0% (*n* = 1209) were current smokers, 14.3% (*n* = 909) reported current alcohol consumption, and 37.5% (*n* = 2026 and 358, respectively) often or always consumed pickled foods. The mean household salt intake was 618.8 ± 364.0 g/month. Anthropometric measurements revealed a mean waist circumference of 89.0 ± 9.6 cm and hip circumference of 96.9 ± 7.8 cm. BMI distribution indicated that 27.6% (*n* = 1751) fell within the normal range (18.5–23.9 kg/m^2^), while 41.0% (*n* = 2604) and 29.0% (*n* = 1902) were categorized as overweight (23.9–27.9 kg/m^2^) or obese (> 27.9 kg/m^2^), respectively. Medical histories showed that 18.2% (*n* = 1159) had major cardiovascular disease (including hyperlipidemia, myocardial infarction, stroke, or HF), 8.4% (*n* = 535) had diabetes, and 0.6% (*n* = 36) reported CKD. Antihypertensive medication use was predominantly monotherapy (86.3%, *n* = 5484), with a mean monthly hypertension-related expenditure of CNY 38.2 ± 39.0. Participants demonstrated limited hypertension knowledge, with a mean score of 5.3 ± 3.0. The average SBP and DBP of the participants were 157.3 ± 20.2 mmHg and 87.5 ± 11.6 mmHg, respectively.

**Figure 1:**
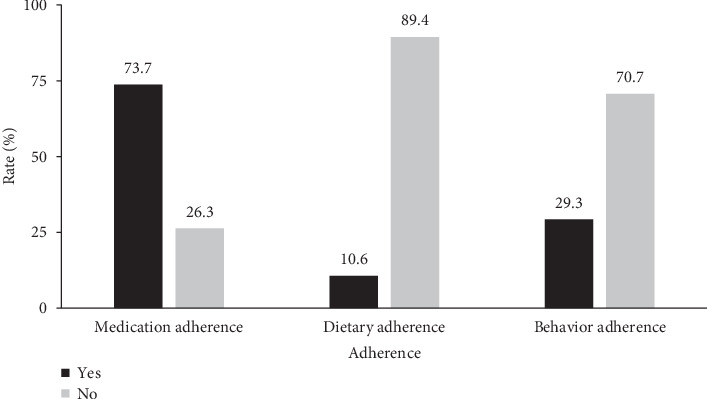
Participants' medication adherence, diet adherence, and behavior adherence rate.

Regarding adherence, 73.7% (*n* = 4679) reported medication adherence, while dietary and behavioral adherence rates were notably lower at 10.5% (*n* = 670) and 29.3% (*n* = 1863), respectively. Participants' medication adherence, diet adherence, and behavior adherence rates are shown in Figure 1. Detailed demographic and clinical characteristics stratified by adherence categories are presented in Table 2.

### 3.2. Univariate Analysis

The results of univariate logistic regression analyses are summarized in Table 2. For medication adherence, significant associations were observed with the following variables: ethnicity (non-Han ethnicities vs. Han, cOR = 0.457, *p* < 0.001), alcohol consumption (former drinkers vs. never drink, cOR = 1.459, *p*=0.003), frequent pickled food intake (always vs. never, cOR = 1.469, *p*=0.015), household monthly salt intake (per gram increase, cOR = 1.000, *p*=0.036), fruit/vegetable consumption (2–3 kg/week vs. seldom, cOR = 1.454, *p*=0.007), physical activity (3–4 times/week vs. never, cOR = 1.237, *p*=0.020), BMI (> 27.9 vs. 18.5–23.9, cOR = 1.267, *p*=0.002), history of major cardiovascular disease (cOR = 0.663, *p* < 0.001), history of diabetes (cOR = 1.274, *p*=0.025), antihypertensive medication use (2 vs. 1 type daily, cOR = 1.542, *p* < 0.001), monthly hypertension-related expenditure (per CNY increase, cOR = 0.998, *p*=0.007), and hypertension knowledge score (per unit increase, cOR = 1.043, *p* < 0.001).

For dietary adherence, significant predictors included household composition (2 vs. 1 members, cOR = 1.958, *p*=0.002; ≥ 3 vs. 1 members, cOR = 3.998, *p* < 0.001), residential status (live with children vs. living alone or with spouse only, cOR = 2.187, *p* < 0.001), education (primary school vs. not educated, cOR = 0.808, *p*=0.041), marital status (divorce/live apart/single vs. married/cohabiting, cOR = 0.299, *p*=0.04), cigarette smoking (current smokers vs. never smoked, cOR = 0.645, *p* < 0.001), alcohol consumption (current drinker vs. never drink, cOR = 0.671, *p*=0.002), physical activity (1–2 times/week vs. never, cOR = 0.492, *p* < 0.001; 3–4 times/week vs. never, cOR = 0.454, *p* < 0.001; 5–6 times/week vs. never, cOR = 0.587, *p* < 0.001), BMI (23.9–27.9 vs. 18.5–23.9, cOR = 1.285, *p*=0.019; > 27.9 vs. 18.5–23.9, cOR = 1.516, *p* < 0.001), antihypertensive medication use (2 vs. 1 type daily, cOR = 1.356, *p*=0.008), and hypertension knowledge score (per unit increase, cOR = 1.04, *p*=0.003).

For behavioral adherence, significant associations were identified with age (≥ 65 vs. < 65, cOR = 0.495, *p* < 0.001), sex (female vs. male, cOR = 3.463, *p* < 0.001), household composition (2 vs. 1 members, cOR = 1.506, *p* < 0.001; ≥ 3 vs. 1 member, cOR = 1.675, *p* < 0.001), residential status (live with children vs. living alone or with spouse only, cOR = 1.12, *p*=0.048), marital status (widower/widow vs. married/cohabiting, cOR = 0.629, *p* < 0.001), frequent pickled food intake (always vs. never, cOR = 1.583, *p* < 0.001), fruit/vegetable intake (2–3 kg/week vs. seldom, cOR = 1.448, *p*=0.011; ≥ 4 kg/week vs. seldom, cOR = 1.75, *p* < 0.001), BMI (23.9–27.9 vs. 18.5–23.9, cOR = 1.373, *p* < 0.001; > 27.9 vs. 18.5–23.9, cOR = 1.694, *p* < 0.001), hipline (per cm increase, cOR = 1.01, *p*=0.006), history of major cardiovascular disease (cOR = 1.456, *p* < 0.001), and antihypertensive medication use (2 vs. 1 type daily, cOR = 1.282, *p*=0.002).

### 3.3. The Associated Factors of Medication Adherence, Dietary Adherence, and Behavior Adherence

#### 3.3.1. Medication Adherence

The results of the goodness-of-fit test indicated that the constructed model exhibited a satisfactory fit (*χ*^2^ = 3.985, *p*=0.859), while the findings from the model hypothesis test demonstrated statistical significance (*χ*^2^ = 191.604, *p* < 0.001). Diagnostic assessments for multicollinearity revealed an absence of multicollinearity among the independent variables (all VIF < 5.0). Multivariate logistic regression analysis identified several factors significantly associated with medication adherence among hypertensive patients (Table 3). Ethnicity demonstrated a notable impact, with Han Chinese patients exhibiting significantly lower adherence compared to other ethnic groups (aOR = 0.485, 95% CI: 0.377–0.624; *p* < 0.001). Alcohol consumption patterns revealed that former drinkers had higher adherence than never drinkers (aOR = 1.393, 95% CI: 1.079–1.797; *p*=0.011), while current drinkers showed no significant difference. Frequent consumption of pickled food (“always”) was positively associated with adherence (aOR = 1.405, 95% CI: 1.022–1.933; *p*=0.036), whereas occasional or moderate intake did not reach statistical significance. Fruit and vegetable consumption of less than 2 kg/week correlated with reduced adherence (aOR = 0.728, 95% CI: 0.546–0.969; *p*=0.03). Physical activity 3–4 times/week was linked to better adherence (aOR = 1.276, 95% CI: 1.061–1.536; *p*=0.01), though no significant trends emerged for other frequencies. A history of major cardiovascular disease inversely impacted adherence (aOR = 0.711, 95% CI: 0.617–0.819; *p* < 0.001). Patients prescribed two types of antihypertensive medications exhibited higher adherence than those on monotherapy (aOR = 1.605, 95% CI: 1.324–1.945; *p* < 0.001). Higher monthly hypertension-related expenditure marginally reduced adherence (aOR = 0.997, 95% CI: 0.996–0.999; *p*=0.001). Conversely, greater hypertension knowledge scores were positively associated with adherence (aOR = 1.033, 95% CI: 1.013–1.052; *p*=0.001).

#### 3.3.2. Dietary Adherence

The associated factors of dietary adherence are shown in Table 4. The results of the goodness-of-fit test indicated that the constructed model exhibited a satisfactory fit (*χ*^2^ = 5.676, *p*=0.683), while the findings from the model hypothesis test demonstrated statistical significance (*χ*^2^ = 182.619, *p* < 0.001). Diagnostic assessments for multicollinearity revealed an absence of multicollinearity among the independent variables (all VIF < 5.0). Multivariate logistic regression analysis revealed significant associations between dietary adherence and sociodemographic, behavioral, and knowledge-based factors among hypertensive patients. Residential status markedly influenced adherence, with patients living with children demonstrating over twofold higher odds of adherence compared to those living alone or with a spouse only (aOR = 2.184, 95% CI: 1.854–2.573; *p* < 0.001). Marital status also played a role: widowed individuals exhibited reduced adherence relative to married/cohabiting counterparts (aOR = 0.698, 95% CI: 0.528–0.924; *p*=0.012). Former smokers displayed higher adherence than never smokers (aOR = 1.525, 95% CI: 1.095–2.123; *p*=0.012). Alcohol consumption, both former (aOR = 0.650, 95% CI: 0.430–0.984; *p*=0.042) and current (aOR = 0.700, 95% CI: 0.517–0.948; *p*=0.021), was inversely associated with adherence. Notably, higher physical activity frequency correlated with reduced adherence. For instance, exercising 3–4 times/week yielded significantly lower odds (aOR = 0.440, 95% CI: 0.333–0.582; *p* < 0.001), with similar trends for 1–2 times/week (aOR = 0.503; *p* < 0.001) and 5–6 times/week (aOR = 0.589; *p* < 0.001). Hypertension knowledge emerged as a positive predictor, with each unit increase in knowledge score associated with a 3.5% higher likelihood of adherence (aOR = 1.035, 95% CI: 1.008–1.063; *p*=0.012).

#### 3.3.3. Behavior Adherence

Table 5 presents the results of the multivariable logistic regression analysis for behavior adherence. The results of the goodness-of-fit test indicated that the constructed model exhibited a satisfactory fit (*χ*^2^ = 4.320, *p*=0.319), while the findings from the model hypothesis test demonstrated statistical significance (*χ*^2^ = 637.449, *p* < 0.001). Diagnostic assessments for multicollinearity revealed an absence of multicollinearity among the independent variables (all VIF < 5.0). Age demonstrated a significant inverse relationship, with patients aged ≥ 65 years exhibiting 42% lower odds of adherence compared to those < 65 years (aOR = 0.58, 95% CI: 0.511–0.659; *p* < 0.001). Females showed substantially higher adherence than males (aOR = 3.596, 95% CI: 3.132–4.128; *p* < 0.001). Educational attainment also influenced adherence, with middle school or higher education associated with a 27.8% increase in adherence odds compared to no formal education (aOR = 1.278, 95% CI: 1.069–1.528; *p*=0.007). Marital status further differentiated adherence patterns: widowed individuals had 27.4% lower odds of adherence relative to married/cohabiting patients (aOR = 0.726, 95% CI: 0.595–0.887; *p*=0.002). Frequent consumption of pickled food (“always”) correlated with 48.9% higher adherence odds (aOR = 1.489, 95% CI: 1.109–1.999; *p*=0.008), while fruit/vegetable intake ≥ 4 kg/week was linked to a 59.8% increase in adherence (aOR = 1.598, 95% CI: 1.198–2.131; *p*=0.001). Patients with a history of major cardiovascular disease exhibited 49.5% higher adherence odds (aOR = 1.495, 95% CI: 1.295–1.725; *p* < 0.001). Use of two antihypertensive medications was associated with a 25.3% adherence increase compared to monotherapy (aOR = 1.253, 95% CI: 1.058–1.484; *p*=0.009). Higher hypertension knowledge scores were positively associated with behavior adherence (aOR = 1.027, 95% CI: 1.008–1.045; *p*=0.005).

## 4. Discussion

Optimal adherence to therapeutic regimens is a critical determinant of effective blood pressure control, complication mitigation, and quality-of-life enhancement in hypertensive populations. These findings revealed that 73.7% of participants demonstrated positive medication adherence—a rate marginally lower than the 77.2% reported by Liu et al. [36] in a comparable cohort but higher than adherence levels documented in studies from other Asian countries and regions [11, 37, 38]. These discrepancies may stem from methodological variations in adherence assessment. Unlike the Morisky Medication Adherence Scale (MMAS-4), which evaluates adherence cessation upon symptom improvement, our criteria did not incorporate this dimension, potentially inflating adherence estimates relative to standardized tools. Notably, only 10.5% of participants exhibited satisfactory dietary adherence, a proportion substantially lower than rates reported in prior Chinese studies [39, 40]. This divergence may reflect the entrenched culinary practices of Northeast China, where diets are characteristically high in sodium and pickled foods—a cultural preference exacerbated by regional climate and food preservation traditions. Such dietary patterns likely undermine compliance with low-sodium recommendations, underscoring the need for culturally tailored nutritional interventions. In contrast, 29.3% of participants demonstrated favorable behavioral adherence, as evidenced by abstaining from smoking, refraining from alcohol consumption, and engaging in regular and adequate physical exercise. A study conducted in the United States on adherence to healthy lifestyle behaviors among adult hypertensive patients reported that 36.9% of patients were able to adhere to three healthy lifestyle behaviors, a proportion higher than that observed in the present study [41]. These findings collectively emphasize the multifactorial nature of adherence, shaped by sociocultural, clinical, and behavioral determinants. To address modifiable risk factors, prioritized strategies should include community-based health education programs, personalized behavioral counseling, and integration of dietary support systems that account for regional food practices.

The results of this study indicated that ethnicity, alcohol drinking, pickled food intake, fruit and vegetable consumption, physical activity, history of major cardiovascular disease, antihypertensive medication use, monthly hypertension-related expenditure, and hypertension knowledge were significantly associated with medication adherence. Dietary adherence was influenced by residential status, marital status, cigarette smoking, alcohol drinking, physical activity, and hypertension knowledge. Behavior adherence exhibited multifactorial determinants, including age, sex, education, marital status, pickled food, fruit and vegetable consumption, history of major cardiovascular disease, antihypertensive medication use, and hypertension knowledge.

This study found that the Han Chinese population had poorer medication adherence compared to other ethnic groups, a phenomenon that has not been reported in previous research. Prior studies have demonstrated significant differences in hypertension prevalence, awareness, treatment, and control rates between ethnic minority populations and the Han Chinese population [42–44]. This study hypothesizes that the disparity in medication adherence may be influenced by a variety of factors, including cultural differences, accessibility to healthcare resources, and socioeconomic status. The interplay of these factors may further impact patients' adherence to treatment, thereby significantly affecting disease management. This study revealed that participants living with their children demonstrated superior dietary adherence, which may be attributed to enhanced family support. Research by Ma et al. [45] indicates a significant association between adequate family support and reduced salt intake behaviors in children. Cohabiting parents may adopt healthier dietary practices through dual mechanisms: primarily motivated by concern for their offspring's health, they tend to implement nutritional optimization in family meals. Concurrently, they may receive positive feedback from their children regarding these dietary modifications, thereby reinforcing their commitment to maintaining healthy eating patterns. The present investigation further identified that widowed participants exhibited inferior dietary and behavioral adherence compared to their married/cohabiting counterparts, potentially associated with diminished familial and social support systems. Existing research has demonstrated that family-based social support serves as critical determinants influencing adherence, enhancing self-care capacity, and improving the quality of life among hypertensive populations [46]. This study identified diminished behavioral adherence among older patients, potentially attributable to the greater difficulty in modifying entrenched lifestyle habits, particularly smoking and alcohol consumption maintained over extended durations. Furthermore, female participants demonstrated superior behavioral adherence, a finding corroborated by epidemiological investigations documenting significantly higher prevalence rates of smoking and hazardous alcohol use among male hypertensive populations [47, 48]. Empirical evidence further suggests enhanced self-regulatory capacity in females, manifested through elevated self-efficacy expectations regarding health behavior maintenance [49]. Additionally, patients with junior high school or higher educational attainment exhibited significantly better adherence profiles compared to those with no formal education, a finding consistent with Liu et al. [36].

This study identified that hypertensive patients with habitual pickled food consumption exhibited better medication and behavioral adherence, partially aligning with findings from Hu et al. [50]. Although global consensus emphasizes the cardiovascular benefits of reducing sodium-rich foods such as pickled products [51, 52], adherence to sodium restriction remains suboptimal, with only 55.3% of hypertensive patients consistently limiting dietary sodium intake [34]. This discrepancy may reflect a complex interplay between entrenched high-salt dietary habits and health-seeking behaviors. Patients with long-standing preferences for sodium-dense diets often struggle to modify these deeply rooted culinary practices; however, our data suggest that heightened health consciousness within this subgroup may paradoxically drive stricter compliance with pharmacological regimens—a phenomenon calls for further exploration. Furthermore, this study has discovered that participants with weekly intake below 2 kg demonstrated suboptimal medication adherence compared to those with minimal consumption (seldom intake). In contrast, those maintaining moderate (2–3 kg/week) and high (≥ 4 kg/week) intake levels exhibited significantly enhanced behavioral adherence. A well-balanced diet has been demonstrated to mitigate the risk of hypertension and cardiovascular diseases [16]. Dietary Guidelines for Chinese Residents (2022) recommend a daily intake of at least 300 g of vegetables and 200 g of fruits for Chinese adults [35]. The significant association observed between the consumption of vegetables and fruits and adherence may underscore the potential influence of nutritional factors on the self-management behaviors of individuals with hypertension.

This research indicated that former alcohol consumers demonstrated enhanced medication adherence compared to never-users, consistent with the findings of Liu et al. [36]. However, both former and current alcohol users exhibited suboptimal dietary adherence, a phenomenon potentially rooted in the synergistic interplay between entrenched culinary traditions and alcohol-centric social practices prevalent in Northeast China. Notably, former smokers displayed superior dietary adherence, suggesting that smoking cessation may reflect broader health-conscious behavioral adaptations, where individuals proactively modify multiple lifestyle risk factors through heightened self-regulatory capacity. Previous studies have shown that individual health-related behaviors are closely linked to compliance behavior [53, 54]. Quitting smoking and reducing alcohol consumption is also one of the lifestyle interventions explicitly recommended in the 2024 Chinese Guidelines for the Prevention and Treatment of Hypertension [4]. This study further identified a protective association between moderate physical activity and enhanced medication adherence, consistent with findings by Euna Han et al. [54]. Regular exercise was associated not only with improved treatment compliance but also demonstrated significant benefits in blood pressure control and reduced risks of cardiovascular and all-cause mortality [55]. However, this study observed that compared to non-exercising participants, those engaging in physical exercise 1–2, 3–4, or 5–6 times weekly demonstrated reduced dietary adherence. The counterintuitive relationship between exercise frequency and dietary adherence suggests potential behavioral compensation mechanisms, whereby increased physical activity frequency may inadvertently trigger dietary relaxation through psychological licensing effects. The underlying mechanisms warrant further investigation.

As found in other studies [56, 57], this study found that patients with a history of major cardiovascular disease showed poorer medication adherence but better behavioral adherence. Chang et al.'s research on the relationship between behavioral factors and medication non-adherence of hypertension found that patients with related comorbidities had a 3.8 times higher risk of reducing drug dosage than patients without comorbidities [58]. This investigation further demonstrated superior medication and behavioral adherence among patients prescribed two daily antihypertensive agents compared to single-drug regimens, corroborating previous findings [59]. Potential explanatory mechanisms include (1) heightened disease awareness associated with therapeutic complexity, which may enhance perceived illness severity and self-care motivation; (2) increased healthcare contact frequency through medication replenishment visits, creating opportunities for adherence reinforcement through clinical monitoring and health education interventions. Notably, this analysis revealed a significant inverse association between hypertension-related monthly expenditures and medication adherence, despite minimal odds ratio values. This finding aligns with established socioeconomic determinants of treatment compliance, as extensively documented in low-income populations [60–62]. Mechanistically, the financial toxicity induced by sustained hypertension management costs may force economically vulnerable middle-aged/elderly patients in rural regions to prioritize essential living expenses over therapeutic continuity—a phenomenon termed “health expenditure triage [63].” This economic burden likely interacts with symptom-driven adherence patterns, where transient clinical improvement fosters premature treatment discontinuation among resource-constrained individuals. This study conclusively demonstrated that elevated hypertension knowledge scores functioned as independent protective factors for medication, dietary, and behavioral adherence. However, the population's mean knowledge score remained suboptimal, highlighting critical health literacy deficits. Existing evidence supports the effectiveness of health education in improving treatment adherence [56, 64]. The adoption of a more accessible and easier to implement health education mode may be more conducive to the adherence of middle-aged and elderly hypertensive patients in rural areas.

Several limitations merit consideration when interpreting the findings. First, the reliance on self-reported questionnaire-based methodology for adherence evaluation introduces potential measurement inaccuracies. This approach may be susceptible to inherent biases including participant selection bias and retrospective recall bias, potentially leading to an overestimation of adherence rates. The lack of a universally accepted gold standard for adherence assessment, compounded by the multidimensional nature of adherence parameters examined in this investigation, presents substantial methodological challenges in establishing robust quantitative metrics. Second, data collection occurred prior to the COVID-19 pandemic. Emerging evidence suggests pandemic-related lifestyle modifications among elderly hypertensive populations [65], a confounder unaccounted for in the current analysis. Third, despite implementing on-site one-to-one questionnaires with oversight by local village doctors, residual bias in survey administration remains possible. Finally, the cross-sectional design inherently precludes causal inference. Notwithstanding these limitations, this investigation provides a multidimensional evaluation of hypertension adherence across medication, dietary, and behavioral domains, with rigorous adjustment for numerous covariates. The results offer valuable insights for optimizing hypertension management strategies in middle-aged and older populations, particularly in resource-limited settings. Future longitudinal studies incorporating pandemic-era behavioral shifts and expanded dietary metrics are warranted to validate these observations.

## 5. Conclusions

This cross-sectional study highlights significant disparities in adherence patterns among middle-aged and older hypertensive patients in rural Northeast China. While medication adherence was relatively high, dietary and behavioral adherence rates remained alarmingly low. Key determinants of adherence included ethnicity, household composition, socioeconomic factors, and health literacy, with distinct predictors identified across adherence domains. Notably, Han Chinese ethnicity, higher hypertension knowledge, and family cohabitation were associated with improved adherence, whereas advanced age, male sex, and lower education levels negatively impacted adherence. The findings underscore the necessity for culturally tailored, multidimensional interventions targeting dietary habits and lifestyle modifications, particularly in regions with entrenched high-sodium culinary practices. Despite limitations such as self-reporting biases and the cross-sectional design, this study provides critical insights into optimizing hypertension management in resource-limited settings. Future research should incorporate longitudinal assessments and contextualize post-pandemic behavioral shifts to strengthen adherence strategies.

## Figures and Tables

**Table 1:** Definition and measurement of adherence.

Adherence	Questions	Measures
Medication adherence	1. Do you ever forget to take or take less antihypertensive drugs? 2. Sometimes if you feel worse when you take the medicine, do you stop taking it?	The answers to both questions were “no.”

Dietary adherence	1. What is the average monthly salt consumption (in kilograms) in your household? 2. How many permanent members are there in your household? 3. How often do you usually consume pickled foods? 4. How much fresh vegetables and fruit do you consume on average per week (in kilograms)?	The average monthly salt consumption per person is ≤ 150 g [35]. Pickled foods are never or only occasionally consumed. The average weekly intake of vegetables and fruits is ≥ 3 kg [35].

Behavioral adherence	1. How often do you smoke? 2. How often do you consume alcohol? 3. How often do you exercise on average per week?	Do not smoke or have quit smoking. Do not consume alcohol or have quit drinking. Exercise five or more times per week [35].

**Table 2:** Medication, dietary, and behavior in participants with different characteristics.

Characteristics of participants	All participants (*n* = 6352)	Medication adherence		cOR	*p*	Dietary adherence		cOR	*p*	Behavior adherence		cOR	*p*
		No (*n* = 1673)	Yes (*n* = 4679)			No (*n* = 5682)	Yes (*n* = 670)			No (*n* = 4489)	Yes (*n* = 1863)		
Age (years), *n* (%)													
< 65	3512 (55.3)	917 (54.8)	2595 (55.5)	Ref	Ref	3135 (55.2)	377 (56.3)	Ref	Ref	2260 (50.3)	1252 (67.2)	Ref	Ref
≥ 65	2840 (44.7)	756 (45.2)	2084 (44.5)	0.974	0.647	2547 (44.8)	293 (43.7)	0.957	0.59	2229 (49.7)	611 (32.8)	0.495	< 0.001
Sex, *n* (%)													
Male	2371 (37.3)	602 (36.0)	1769 (37.8)	Ref	Ref	2143 (37.7)	228 (34)	Ref	Ref	2016 (44.9)	355 (19.1)	Ref	Ref
Female	3981 (62.7)	1071 (64.0)	2910 (62.2)	0.925	0.186	3539 (62.3)	442 (66)	1.174	0.062	2473 (55.1)	1508 (80.9)	3.463	< 0.001
Ethnicity, *n* (%)													
Han	5828 (91.8)	1596 (95.4)	4232 (90.4)	0.457	< 0.001	5211 (91.7)	617 (92.1)	1.052	0.736	4129 (92)	1699 (91.2)	0.903	0.302
Others	524 (8.2)	77 (4.6)	447 (9.6)	Ref	Ref	471 (8.3)	53 (7.9)	Ref	Ref	360 (8)	164 (8.8)	Ref	Ref
Household composition													
1	560 (8.8)	147 (8.8)	413 (8.8)	Ref	Ref	536 (9.4)	24 (3.6)	Ref	Ref	440 (9.8)	120 (6.4)	Ref	Ref
2	3276 (51.6)	863 (51.6)	2413 (51.6)	0.995	0.963	3012 (53)	264 (39.4)	1.958	0.002	2322 (51.7)	954 (51.2)	1.506	< 0.001
≥ 3	2516 (39.6)	663 (39.6)	1853 (39.6)	0.995	0.961	2134 (37.6)	382 (57)	3.998	< 0.001	1727 (38.5)	789 (42.4)	1.675	< 0.001
Residential status, *n* (%)													
Living alone or with spouse only	3974 (62.6)	1066 (63.7)	2908 (62.2)	Ref	Ref	3666 (64.5)	308 (46)	Ref	Ref	2843 (63.3)	1131 (60.7)	Ref	Ref
Live with children	2287 (36.0)	586 (35.0)	1701 (36.4)	1.064	0.299	1932 (34)	355 (53)	2.187	< 0.001	1582 (35.2)	705 (37.8)	1.12	0.048
Others	91 (1.4)	21 (1.3)	70 (1.5)	1.222	0.425	84 (1.5)	7 (1)	0.992	0.984	64 (1.4)	27 (1.4)	1.06	0.8
Education, *n* (%)													
Not educated	1307 (20.6)	366 (21.9)	941 (20.1)	Ref	Ref	1153 (20.3)	154 (23)	Ref	Ref	934 (20.8)	373 (20)	Ref	Ref
Primary school	3263 (51.4)	857 (51.2)	2406 (51.4)	1.092	0.23	2945 (51.8)	318 (47.5)	0.808	0.041	2324 (51.8)	939 (50.4)	1.012	0.872
Middle school or higher	1782 (28.1)	450 (26.9)	1332 (28.5)	1.151	0.087	1584 (27.9)	198 (29.6)	0.936	0.562	1231 (27.4)	551 (29.6)	1.121	0.153
Marital status, *n* (%)													
Married/cohabiting	5545 (87.3)	1472 (88.0)	4073 (87.0)	Ref	Ref	4940 (86.9)	605 (90.3)	Ref	Ref	3856 (85.9)	1689 (90.7)	Ref	Ref
Widower/widow	722 (11.4)	177 (10.6)	545 (11.6)	1.113	0.244	660 (11.6)	62 (9.3)	0.767	0.058	566 (12.6)	156 (8.4)	0.629	< 0.001
Divorce/live apart/single	85 (1.3)	24 (1.4)	61 (1.3)	0.919	0.727	82 (1.4)	3 (0.4)	0.299	0.04	67 (1.5)	18 (1)	0.613	0.067
Cigarette smoking, *n* (%)													
Never smoked	4689 (73.8)	1267 (75.7)	3422 (73.1)	Ref	Ref	4169 (73.4)	520 (77.6)	Ref	Ref	2946 (65.6)	1743 (93.6)	—	—
Former smokers	454 (7.1)	107 (6.4)	347 (7.4)	1.201	0.113	394 (6.9)	60 (9)	1.221	0.172	334 (7.4)	120 (6.4)	—	—
Current smokers	1209 (19.0)	299 (17.9)	910 (19.4)	1.127	0.108	1119 (19.7)	90 (13.4)	0.645	< 0.001	1209 (26.9)	0 (0)	—	—
Alcohol drinking, *n* (%)													
Never drink	5040 (79.3)	1353 (80.9)	3687 (78.8)	Ref	Ref	4475 (78.8)	565 (84.3)	Ref	Ref	3259 (72.6)	1781 (95.6)	—	—
Former drinker	403 (6.3)	81 (4.8)	322 (6.9)	1.459	0.003	369 (6.5)	34 (5.1)	0.73	0.088	321 (7.2)	82 (4.4)	—	—
Current drinker	909 (14.3)	239 (14.3)	670 (14.3)	1.029	0.729	838 (14.7)	71 (10.6)	0.671	0.002	909 (20.2)	0 (0)	—	—
Pickled food, *n* (%)													
Never	596 (9.4)	169 (10.1)	427 (9.1)	Ref	Ref	501 (8.8)	95 (14.2)	—	—	424 (9.4)	172 (9.2)	Ref	Ref
Occasionally	3372 (53.1)	867 (51.8)	2505 (53.5)	1.144	0.176	2797 (49.2)	575 (85.8)	—	—	2372 (52.8)	1000 (53.7)	1.039	0.694
Often	2026 (31.9)	561 (33.5)	1465 (31.3)	1.034	0.75	2026 (35.7)	0 (0)	—	—	1475 (32.9)	551 (29.6)	0.921	0.425
Always	358 (5.6)	76 (4.5)	282 (6.0)	1.469	0.015	358 (6.3)	0 (0)	—	—	218 (4.9)	140 (7.5)	1.583	0.001
Household monthly salt intake (g/month), (mean ± SD)	618.8 ± 363.96	602.7 ± 334.02	624.5 ± 373.95	1	0.036	647.0 ± 369.77	379.2 ± 179.49	—	—	615.0 ± 346.82	627.8 ± 402.22	1	0.206
Fruit/vegetable consumption (kg/week), *n* (%)													
Seldom	325 (5.1)	95 (5.7)	230 (4.9)	Ref	Ref	325 (5.7)	0 (0)	—	—	253 (5.6)	72 (3.9)	Ref	Ref
< 2 kg	826 (13.0)	280 (16.7)	546 (11.7)	0.805	0.129	826 (14.5)	0 (0)	—	—	629 (14)	197 (10.6)	1.101	0.541
2–3 kg	1374 (21.6)	304 (18.2)	1070 (22.9)	1.454	0.007	1374 (24.2)	0 (0)	—	—	973 (21.7)	401 (21.5)	1.448	0.011
3–4 kg	1231 (19.4)	296 (17.7)	935 (20.0)	1.305	0.056	1020 (18)	211 (31.5)	—	—	901 (20.1)	330 (17.7)	1.287	0.089
≥ 4 kg	2596 (40.9)	698 (41.7)	1898 (40.6)	1.123	0.371	2137 (37.6)	459 (68.5)	—	—	1733 (38.6)	863 (46.3)	1.75	< 0.001
Physical activity, *n* (%)													
Never	1878 (29.6)	481 (28.8)	1397 (29.9)	Ref	Ref	1622 (28.5)	256 (38.2)	Ref	Ref	1878 (41.8)	0 (0)	—	—
1–2 times/week	749 (11.8)	217 (13)	532 (11.4)	0.844	0.079	695 (12.2)	54 (8.1)	0.492	< 0.001	749 (16.7)	0 (0)	—	—
3–4 times/week	1047 (16.5)	228 (13.6)	819 (17.5)	1.237	0.02	977 (17.2)	70 (10.4)	0.454	< 0.001	1047 (23.3)	0 (0)	—	—
5–6 times/week	767 (12.1)	210 (12.6)	557 (11.9)	0.913	0.348	702 (12.4)	65 (9.7)	0.587	< 0.001	240 (5.3)	527 (28.3)	—	—
≥ 7 times/week	1911 (30.1)	537 (32.1)	1374 (29.4)	0.881	0.084	1686 (29.7)	225 (33.6)	0.846	0.086	575 (12.8)	1336 (71.7)	—	—
BMI, *n* (%)													
BMI < 18.5	95 (1.5)	23 (1.4)	72 (1.5)	1.258	0.349	87 (1.5)	8 (1.2)	0.989	0.976	73 (1.6)	22 (1.2)	0.976	0.924
18.5 ≤ BMI ≤ 23.9	1751 (27.6)	502 (30.0)	1249 (26.7)	Ref	Ref	1602 (28.2)	149 (22.2)	Ref	Ref	1338 (29.8)	413 (22.2)	Ref	Ref
23.9 < BMI ≤ 27.9	2604 (41.0)	690 (41.2)	1914 (40.9)	1.115	0.115	2326 (40.9)	278 (41.5)	1.285	0.019	1829 (40.7)	775 (41.6)	1.373	< 0.001
BMI > 27.9	1902 (29.0)	458 (27.4)	1444 (30.9)	1.267	0.002	1667 (29.3)	235 (35.1)	1.516	< 0.001	1249 (27.8)	653 (35.1)	1.694	< 0.001
Waistline (cm), (mean ± SD)	89.0 ± 9.61	89.1 ± 9.61	89.0 ± 9.61	0.998	0.52	89.0 ± 9.65	89.1 ± 9.23	1.001	0.799	89.0 ± 9.96	89.1 ± 9.43	1.002	0.596
Hipline (cm), (mean ± SD)	96.9 ± 7.76	96.9 ± 7.70	96.8 ± 7.78	0.998	0.612	96.8 ± 7.80	97.3 ± 7.35	1.009	0.085	96.7 ± 7.83	97.2 ± 7.57	1.01	0.006
History of major cardiovascular disease^∗^, *n* (%)													
No	5193 (81.8)	1288 (77.0)	3905 (83.5)	Ref	Ref	4649 (81.8)	544 (81.2)	Ref	Ref	3747 (83.5)	1446 (77.6)	Ref	Ref
Yes	1159 (18.2)	385 (23.0)	774 (16.5)	0.663	< 0.001	1033 (18.2)	126 (18.8)	1.042	0.692	742 (16.5)	417 (22.4)	1.456	< 0.001
History of diabetes, *n* (%)													
No	5817 (91.6)	1554 (92.9)	4263 (91.1)	Ref	Ref	5201 (91.5)	616 (91.9)	Ref	Ref	4119 (91.8)	1698 (91.1)	Ref	Ref
Yes	535 (8.4)	119 (7.1)	416 (8.9)	1.274	0.025	481 (8.5)	54 (8.1)	0.948	0.721	370 (8.2)	165 (8.9)	1.082	0.422
History of chronic kidney disease, *n* (%)													
No	6316 (99.4)	1664 (99.5)	4652 (99.4)	Ref	Ref	5652 (99.5)	664 (99.1)	Ref	Ref	4463 (99.4)	1853 (99.5)	Ref	Ref
Yes	36 (0.6)	9 (0.5)	27 (0.6)	1.073	0.855	30 (0.5)	6 (0.9)	1.702	0.236	26 (0.6)	10 (0.5)	0.926	0.838
Antihypertensive medication use, *n* (%)													
1	5484 (86.3)	1499 (89.6)	3985 (85.2)	Ref	Ref	4929 (86.7)	555 (82.8)	Ref	Ref	3917 (87.3)	1567 (84.1)	Ref	Ref
2	770 (12.0)	151 (9.0)	619 (13.2)	1.542	< 0.001	668 (11.8)	102 (15.2)	1.356	0.008	509 (11.3)	261 (14)	1.282	0.002
≥ 3	98 (1.5)	23 (1.4)	75 (1.6)	1.227	0.395	85 (1.5)	13 (1.9)	1.358	0.309	63 (1.4)	35 (1.9)	1.389	0.123
Monthly hypertension-related expenditure (CNY), (mean ± SD)	38.2 ± 39.03	40.4 ± 44.39	37.4 ± 36.9	0.998	0.007	38.2 ± 39.41	38.1 ± 35.65	1	0.975	37.9 ± 38.11	38.8 ± 41.17	1.001	0.42
Hypertension knowledge, (mean ± SD)	5.3 ± 3.04	5 ± 2.97	5.4 ± 3.06	1.043	< 0.001	5.3 ± 3.05	5.7 ± 2.99	1.04	0.003	5.3 ± 3.05	5.3 ± 3.03	0.993	0.438
Average SBP (mmHg), (mean ± SD)	157.3 ± 20.24	156.5 ± 19.91	157.5 ± 20.35	1.003	0.076	157.6 ± 20.31	154.8 ± 19.47	0.993	0.001	157.6 ± 20.36	156.5 ± 19.95	0.997	0.062
Average DBP (mmHg), (mean ± SD)	87.5 ± 11.64	86.7 ± 11.21	87.79 ± 11.78	1.008	0.002	87.6 ± 11.70	86.8 ± 11.09	0.994	0.109	87.4 ± 11.88	87.8 ± 11.05	1.004	0.138

^∗^Major cardiovascular disease includes hyperlipidemia, myocardial infarction, stroke, and heart failure.

**Table 3:** Associated factors of medication adherence.

Associated factors	Coefficient	*p* value	OR	95% CI
Intercept	2.167	< 0.001	—	—
Ethnicity				
Han	−0.723	**< 0.001**	0.485	(0.377–0.624)
Others	Ref	Ref	Ref	Ref
Alcohol drinking				
Never drink	Ref	Ref	Ref	Ref
Former drinker	0.331	**0.011**	1.393	(1.079–1.797)
Current drinker	0.084	0.318	1.088	(0.922–1.283)
Pickled food				
Never	Ref	Ref	Ref	Ref
Occasionally	0.079	0.437	1.082	(0.887–1.321)
Often	−0.05	0.643	0.952	(0.772–1.173)
Always	0.34	**0.036**	1.405	(1.022–1.933)
Fruit/vegetable consumption (kg/week)				
Seldom	Ref	Ref	Ref	Ref
< 2 kg	−0.318	**0.03**	0.728	(0.546–0.969)
2–3 kg	0.222	0.117	1.248	(0.946–1.648)
3–4 kg	0.105	0.46	1.111	(0.841–1.467)
≥ 4 kg	0.019	0.886	1.019	(0.786–1.321)
Physical activity				
Never	Ref	Ref	Ref	Ref
1–2 times/week	−0.101	0.306	0.904	(0.745–1.097)
3–4 times/week	0.244	**0.01**	1.276	(1.061–1.536)
5–6 times/week	−0.09	0.365	0.914	(0.751–1.111)
≥ 7 times/week	−0.106	0.164	0.899	(0.774–1.044)
History of major cardiovascular disease^∗^				
No	Ref	Ref	Ref	Ref
Yes	−0.342	**< 0.001**	0.711	(0.617–0.819)
Antihypertensive medication use				
1	Ref	Ref	Ref	Ref
2	0.473	**< 0.001**	1.605	(1.324–1.945)
≥ 3	0.35	0.157	1.419	(0.874–2.302)
Monthly hypertension-related expenditure (CNY)	−0.003	**0.001**	0.997	(0.996–0.999)
Hypertension knowledge	0.032	**0.001**	1.033	(1.013–1.052)

*Note:* The bold values indicate the statistically significant differences.

^∗^Major cardiovascular disease includes hyperlipidemia, myocardial infarction, stroke, and heart failure.

**Table 4:** Associated factors of dietary adherence.

Associated factors	Coefficient	*p* value	OR	95% CI
Intercept	−2.256	< 0.001	—	—
Residential status				
Living alone or with spouse only	Ref	Ref	Ref	Ref
Live with children	0.781	**< 0.001**	2.184	(1.854–2.573)
Others	0.073	0.856	1.076	(0.488–2.372)
Marital status				
Married/cohabiting	Ref	Ref	Ref	Ref
Widower/widow	−0.359	**0.012**	0.698	(0.528–0.924)
Divorce/live apart/single	−0.894	0.134	0.409	(0.127–1.317)
Cigarette smoking				
Never smoked	Ref	Ref	Ref	Ref
Former smokers	0.422	**0.012**	1.525	(1.095–2.123)
Current smokers	−0.217	0.119	0.805	(0.613–1.057)
Alcohol drinking				
Never drink	Ref	Ref	Ref	Ref
Former drinker	−0.43	**0.042**	0.65	(0.43–0.984)
Current drinker	−0.356	**0.021**	0.7	(0.517–0.948)
Physical activity				
Never	Ref	Ref	Ref	Ref
1–2 times/week	−0.687	**< 0.001**	0.503	(0.369–0.686)
3–4 times/week	−0.821	**< 0.001**	0.44	(0.333–0.582)
5–6 times/week	−0.53	**< 0.001**	0.589	(0.44–0.787)
≥ 7 times/week	−0.162	0.107	0.851	(0.699–1.036)
Hypertension knowledge	0.034	**0.012**	1.035	(1.008–1.063)

*Note:* The variables household composition, pickled food, household monthly salt intake, and fruit/vegetable consumption were excluded from the model as they were utilized to assess dietary adherence. The bold values indicate the statistically significant differences.

**Table 5:** Associated factors of behavior adherence.

Associated factors	Coefficient	*p* value	OR	95% CI
Intercept	−2.627	< 0.001	—	—
Age (years)				
< 65	Ref	Ref	Ref	Ref
≥ 65	−0.544	**< 0.001**	0.58	(0.511–0.659)
Sex				
Male	Ref	Ref	Ref	Ref
Female	1.28	**< 0.001**	3.596	(3.132–4.128)
Education				
Not educated	Ref	Ref	Ref	Ref
Primary school	0.116	0.134	1.123	(0.965–1.307)
Middle school or higher	0.246	**0.007**	1.278	(1.069–1.528)
Marital status				
Married/cohabiting	Ref	Ref	Ref	Ref
Widower/widow	−0.32	**0.002**	0.726	(0.595–0.887)
Divorce/live apart/single	0.189	0.505	1.209	(0.692–2.109)
Pickled food				
Never	Ref	Ref	Ref	Ref
Occasionally	−0.038	0.717	0.963	(0.786–1.181)
Often	−0.136	0.216	0.873	(0.704–1.082)
Always	0.398	**0.008**	1.489	(1.109–1.999)
Fruit/vegetable consumption (kg/week)				
Seldom	Ref	Ref	Ref	Ref
< 2 kg	0.101	0.537	1.107	(0.802–1.527)
2–3 kg	0.337	**0.029**	1.4	(1.035–1.893)
3–4 kg	0.212	0.174	1.236	(0.911–1.677)
≥ 4 kg	0.469	**0.001**	1.598	(1.198–2.131)
History of major cardiovascular disease^∗^				
No	Ref	Ref	Ref	Ref
Yes	0.402	**< 0.001**	1.495	(1.295–1.725)
Antihypertensive medication use				
1	Ref	Ref	Ref	Ref
2	0.226	**0.009**	1.253	(1.058–1.484)
≥ 3	0.341	0.131	1.406	(0.903–2.19)
Hypertension knowledge	0.027	**0.005**	1.027	(1.008–1.045)

*Note:* The variables cigarette smoking, alcohol drinking, and physical activity were excluded from the model as they were utilized to assess behavior adherence. The bold values indicate the statistically significant differences.

^∗^Major cardiovascular disease includes hyperlipidemia, myocardial infarction, stroke, and heart failure.

## Data Availability

The data that support the findings of this study are available from the Ministry of Science and Technology of China. Restrictions apply to the availability of these data, which were used under license for this study. Data are available from the author(s) with the permission of the Ministry of Science and Technology of China.
